# Prehospital trauma care reduces mortality. Ten-year results from a time-cohort and trauma audit study in Iraq

**DOI:** 10.1186/1757-7241-20-13

**Published:** 2012-02-03

**Authors:** Mudhafar K Murad, Stig Larsen, Hans Husum

**Affiliations:** 1 Trauma Care Foundation Iraq, Suleimaniah, Iraq; 2 Institute of Clinical Medicine, Faculty of Health Sciences, University of Tromso, Norway; 3 Center for Epidemiology and Biostatistics, Norwegian School of Veterinary Science, Oslo, Norway; 4 Tromso Mine Victim Resource Center, University Hospital North Norway, Tromso, Norway

**Keywords:** Iraq, Land mine, Life support, Prehospital, Severity indices, Trauma audit, Trauma mortality, War

## Abstract

**Background:**

Blunt implementation of Western trauma system models is not feasible in low-resource communities with long prehospital transit times. The aims of the study were to evaluate to which extent a low-cost prehospital trauma system reduces trauma deaths where prehospital transit times are long, and to identify specific life support interventions that contributed to survival.

**Methods:**

In the study period from 1997 to 2006, 2,788 patients injured by land mines, war, and traffic accidents were managed by a chain-of-survival trauma system where non-graduate paramedics were the key care providers. The study was conducted with a time-period cohort design.

**Results:**

37% of the study patients had serious injuries with Injury Severity Score ≥ 9. The mean prehospital transport time was 2.5 hours (95% CI 1.9 - 3.2). During the ten-year study period trauma mortality was reduced from 17% (95% CI 15 -19) to 4% (95% CI 3.5 - 5), survival especially improving in major trauma victims. In most patients with airway problems, in chest injured, and in patients with external hemorrhage, simple life support measures were sufficient to improve physiological severity indicators.

**Conclusion:**

In case of long prehospital transit times simple life support measures by paramedics and lay first responders reduce trauma mortality in major injuries. Delegating life-saving skills to paramedics and lay people is a key factor for efficient prehospital trauma systems in low-resource communities.

## Introduction

The epidemic of trauma is accelerating. Injury is now the fourth leading cause of global deaths, and up to 2030 WHO estimates a further 40% increase in trauma fatalities. Almost 90% of injury deaths occur in low- and middle-income countries [1]. Who is to manage this heavy load of trauma - in disastrous events as well as chronic emergencies like the land mine epidemic? Studies of Western trauma scenarios consistently report that reduced prehospital transport times and level I trauma centers and are the essential components of a good trauma system [2]. However, helicopter evacuations and high-cost surgical centers are not feasible in low-income societies and in countries where the social fabric is broken by war. In our time, local wars and natural disasters especially hit low-resource communities and here the "scoop-and run-for-the hospital" strategy hardly fits. There is thus an urgent need to develop trauma system models and identify the crucial measures to improve survival in such scenarios. Surveys of post-invasion deaths in Iraq estimate an excess death proportion as a consequence of war corresponding to 2.5% of the population, gunfire and bomb blasts being the most common causes of death [3]. Iraq thus represents a challenging testing ground for new rescue system models.

The aims of the study were to evaluate to which extent a low-cost prehospital trauma system reduces deaths where out-of-hospital times are long, and to identify specific prehospital life support interventions that enhance survival.

## Materials and methods

### Study design

**Figure 1:**
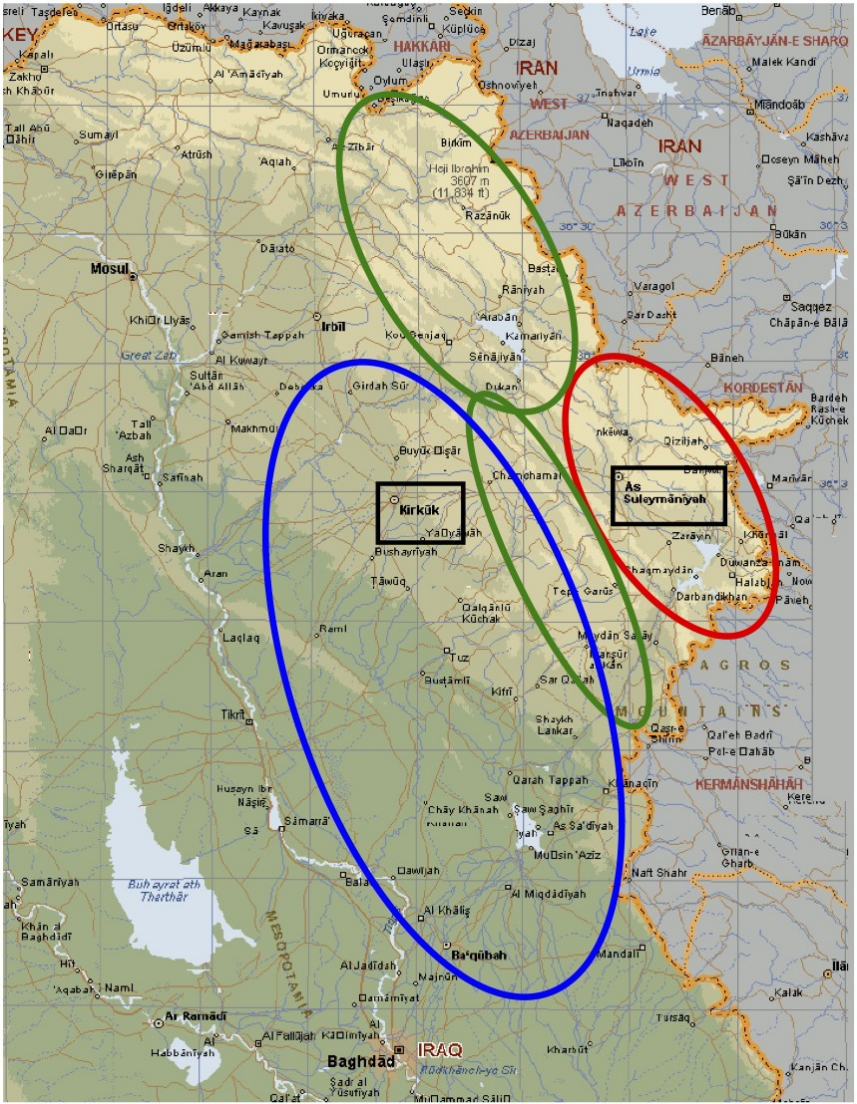
**Trauma system expansion by time periods**. In period 1 (1997 - 2000, red), the trauma system targeted landmine accidents; in period 2, (2001 - 2003, green), the system was expanded to also include highway road-traffic accidents; in period 3 (2004 - 2006, blue) the system additionally focused on war victims. The referral hospitals (Kirkuk and Suleimaniah Teaching Hospitals) are marked in boxes.

The reference population consists of trauma patients in low-income countries with long pre-hospital transport times. The study was conducted with a time-period cohort design defined by a stepwise expansion of the actual trauma system: In period 1, from 1997 to 2000, the catchments area of the prehospital trauma system was the rural mine fields of Northern Iraq; in period 2, from 2001 to 2003, the trauma system was expanded to also target highway traffic accidents in the Northern sector while still being operational in the rural North; from 2004 to 2006 the trauma system developed further to include the war zones of Central Iraq, yet still in action in the previous catchments areas (Figure 1).

### Intervention

The chain-of-survival for prehospital trauma management comprises of three elements: lay trauma first responders at village level, trained paramedics at rural health centers, and emergency room staff at referral hospitals. The actual trauma system was established in 1997 on request from the health authorities in the Kurdistan region of Iraq to rescue land mine and war victims from the vast mine fields along the Iran-Iraqi border. Pre-intervention surveys documented mine casualty mortality at 40%, a figure in accordance with surveys from other mine-infested countries [4]. The paramedics at rural health centers were trained by the authors to provide prehospital trauma life support on-site and during protracted evacuations (table 1). In order to reduce in-field response times and empower the local communities, the paramedics were also trained to teach basic life support measures to laypersons in their area. The training of village first-helpers was done in two-day courses in the villages, targeting men, women and children [5]. Since the invasion of Iraq in 2003 the trauma system was expanded to the war zones of Baquba and Kirkuk and also Emergency Room paramedics at district hospitals and referral centers were included for training. By 2006 the trauma system comprised of 135 paramedics and 7,000 layperson first helpers supervised by six medical doctors. Suleimaniah and Kirkuk Teaching Hospitals were referral centers throughout the study period (Figure 1).

**Table 1:** Pre-hospital treatment protocol

Airway	Breathing	Circulation	Drugs
Head tilt - chin lift. Oro-pharyngeal airway. Suction. Recovery position. Endotracheal intubation/crico-thyrotomy. Cricoid pressure. Heimlich maneuver for choking. Stabilization of neck & spinal cord injuries.	Rescue breathing/CPR. Half-sitting position. Gastric tube decompression. Needle decompression of tension pneumothorax. IV analgesia.	External bleeds: proximal artery compression + sub-fascial packing + compressive dressing + splinting of fractures. Pelvic bleeds: external compression of abdominal aorta. Hypothermia prevention, warming. External jugular cannulation. Venous cut-down. Hypotensive IV fluid resuscitation.	Ketamine. Pentazocine. Atropine. Diazepam. Penicillin. Ampicillin. Metronidazole.

### Data collection and processing

**Figure 2:**
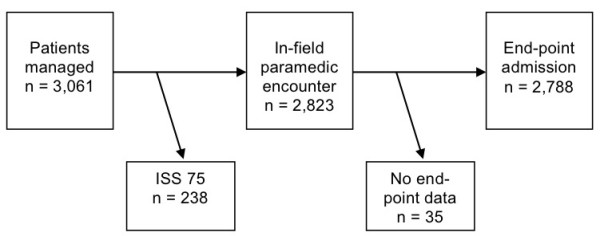
**Study patient flow chart**. Injuries rated at ISS = 75 are not compatible with survival and patients with this rating were excluded from study. End-point data could not be gathered in patients evacuated to surgical centers outside the study area, and these cases were also excluded from study.

All trauma patients managed by the system from January 1997 through December 2006 were consecutively included in a trauma registry. The data were collected at the first in-field encounter with a trained paramedic, and on admission at the referral hospital. The paramedics registered demographic factors, in-field response time from injury to the first encounter with the paramedic, and total prehospital transit time. They also registered physiological indicators at the first encounter in-field and again on hospital admission. All in-field data including photos were scrutinized by the main author at monthly meetings. The data for anatomical severity grading, Injury Severity Score (ISS), were collected by the trauma system supervisors at the referral surgical centers [6]. Due to the local cultural tradition, autopsies on fatal cases were not performed. The main outcome variable was trauma death defined as on-site deaths, deaths during the pre hospital phase, or trauma-related in-hospital deaths. The ISS ranges from 1, light injury, to 75, cases with ISS > 9 being defined as serious, and ISS > 15 as major trauma victims. By definition, patients with ISS = 75 have injuries incompatible with survival, and this subset (n = 238) was excluded from analysis. End-point data could not be collected in 35 patients evacuated to surgical centers outside the study area or cross-border to Iran; also these patients were excluded from the study (Figure 2).

The Physiological Severity Score (PSS) was used for estimation of physiological severity. The PSS is a simplified version of the Revised Trauma Score for triage (RTS) where the Glasgow Coma Scale element is replaced with a five-grade conscious level indicator [7]. The two other indicators, respiratory rate and systolic blood pressure, were rated according to the standard RTS guideline [8]. The PSS score ranges from 0, lifeless, to 12, normal physiological condition. The PSS on admission were compared to the PSS at the first in-field encounter; cases with negative ΔPSS were defined as prehospital treatment failures. Tests of inter-rater reliability in PSS scoring were not undertaken. Audit of patients with unexpected outcomes is an established method of trauma system quality assurance [9]. To identify and review unexpected survivors and unexpected fatalities, a model of death risk prediction was constructed based on the study data. Unexpected survivors were defined as survivors with predicted probability of trauma death (Pd) ≥ 0.5. Unexpected fatalities were defined by two criteria: Pd < 0.25, and in-field PSS ≥ 6.

### Data analysis

Assumed continuously and symmetrically distributed variables are expressed by mean values with 95% confidence intervals (95% CI) constructed by the Student procedure. Due to the irregular shape of several continuous variables, comparisons were undertaken using nonparametric methods [10]. Proportions were described using the exact 95% calculated confidence interval [11]. Receiver Operating Characteristics (ROC) analysis was used to estimate the accuracy of mortality predictors. A predictor is considered accurate if the area under the ROC curve (ROC-AUC) is larger than 0.8 [12]. Most probabilistic models reported in the literature for estimation of trauma mortality risks are based on urban cohorts managed by advanced trauma systems. To develop a risk predictor with optimal fit in the actual study cohort, a logistic regression model was used to identify patients with unexpected outcome. All assumed predictors of trauma death were included using a backward selection process with inclusion at significance level of 5%. The logistic model was evaluated using the Hosmer-Lemeshow test and ROC analysis.

### Ethical considerations

The Directorate of Health Suleimaniah, Ministry of Health, Kurdistan Region gave ethical approval for the study (Ref. no. 22082). There is no other authorized committee for medical research ethics in North Iraq.

The data were stored and processed according to ethical permission from the Norwegian Social Science Data Service (ref. no. 2006/13702).

## Results

The system managed a total of 2,778 patients with mean age of 26 years; there were 22.5% female patients and 22.5% children. The mean ISS was 6.1; 1,034 had injuries with ISS ≥ 9; of these there were 339 major trauma victims. The mean prehospital transit time was 2.5 hours (95% CI: 1.9- 3.2) while 448 victims had evacuation times of more than four hours. The extremity injuries counted for 34% of the total sample while 24% of all patients had critical area injuries (injury to the head, neck, or torso). Most injuries were blunt (71%)(table 2).

**Table 2:** Distribution of injuries by diagnosis with respective mortality rates, 95% confidence intervals for rates given in brackets

	Blunt	Penetrating	Total
**Superficial**	516	109	625
	0%	0%	0%

**Burn**	273	-	273
	15.7% (11.4 - 20.1)		15.7% (11.4 - 20.1)

**Extremities**	567	375	942
	0.7% (0.02 - 1.8)	1.3% (0.2 - 2.5)	0.9% (5.3 - 9.2)

Critical area*	478	194	672
	4.6% (3.1 - 6.9)	12.9% (8.2 - 17.6)	6.9% (5.3 - 9.2)

**Multiple major**	139	127	266
	15.6% (9.4 - 21.7)	43.3% (34.7 - 52.0)	29% (23.5 - 34.5)

**Total**	1,973	805	2,778
	4.5% (3.6 - 5.5)	10.5% (8.4 - 12.6)	6.3% (5.4 - 7.2)

* Critical area: Head, neck, or torso

The overall mortality rate during the study period was 6.3%. The mortality rate differed significantly by body region, being highest for burns and multiple major trauma and significantly higher in penetrating than blunt injuries (table 2). The anatomical and physiological injury severity was higher in the group of non-survivors; no significant differences were observed for other assumed explanatory variables (table 3). Out of 175 prehospital deaths, 75 occurred on-site before the first in-field contact with the paramedic while 23 patients died in the hands of the prehospital care provider. There were 77 in-hospital deaths, 37 of them being burn cases. Of the burn fatalities, 86% occurred more than 48 hours after hospital admission compared to 30% in non-burn fatalities. The mortality rate was significantly higher in female burn victims, 22.5%, compared to male victims, 8% (95% CI diff: 6.5 - 23).

**Table 3:** Comparison between the groups of survivors and non-survivors of assumed explanatory variables for trauma death.

	Survivors (n = 2,613)	Non-survivors (n = 175)
**Age (years)**	26 (25 - 26.8)	27 (24.5 - 29)

**ISS**	6.1 (5.9 - 6.3)	28.7 (27.3 - 30.2)

**PSS-1**	10.1 (10 - 10.2)	4.2 (3.6 - 4.7)

**PSS-2**	11.5 (11.5 - 11.6)	9.1 (8.5 - 9.8)

**In-field response time (hours)**	0.8 (0.7 - 0.9)	1.3 (1.0 - 1.5)

**Total evacuation time (hours)**	2.9 (2.7 - 3.0)	2.5 (1.3 - 3.2)

The results are expressed as means with 95% confidence intervals

### Trauma system outcomes by time cohorts

**Figure 3:**
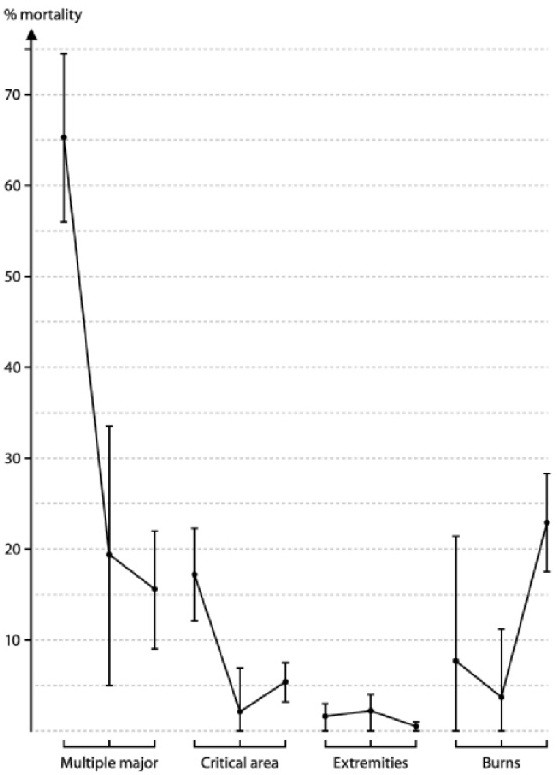
**Mortality rate variations by the three time cohorts**. The estimates are given with 95% confidence interval bars and demonstrate significant reductions in mortality for Multiple Major and Critical Area injuries (injuries to the head, neck, or torso). The mortality rate in burns increased from period 2 to period 3.

The epidemiology of trauma shifted during the study period with a massive increase in the numbers of road traffic casualties in period 3 (table 4). There was a reduction in overall mortality from 17% in period 1 (95% CI: 15 - 19) to 4% in period 2 and 3 (95% CI: 3.5 - 5). Prehospital mortality rates were reduced from 16% in period 1 to 1.7% and 1.3% in period 2 and 3 (95% CI diff: 11 - 18). The main contributions to improved survival were reduced mortality in critical area and multiple major injured. In burn patients the mortality increased from period 2 to period 3 (95% CI diff:11.6% - 26.8%)(Figure 3). Due to reduction of mean injury severity from time cohort 1 onwards (table 5), regression analysis was used to adjust for severity variations. A model combining ISS, PSS and the time cohorts explained 70% of the variations in trauma mortality, ISS being the heaviest predictor with ROC-AUC value > 95%, but also time cohorts contributing significantly. The in-field response times were reduced from 1.6 hours in period 1 to 0.7 hours in period 3 (95% CI diff: 0.7- 1.1), and there was a reduction in total out-of-hospital time from 4.4 hours to 2.3 hours (95% CI diff: 1.8 - 2.4).

**Table 4:** Distribution of injury mechanism by time cohorts, numbers expressed by row percentages

	Time period 1	Time period 2	Time period 3
**Casualties from mines and**	268	209	329
**actions of war**	33%	26%	41%

**Road traffic accident**	27	115	1153
**casualties**	3%	8%	89%

**Burn casualties**	26	83	164
	10%	30%	60%

**Other trauma casualties**	84	64	266
	20%	16%	64%

**Total**	405	471	1912

**Table 5:** Distribution of assumed explanatory variables for trauma death by time cohorts.

	Time period 1	Time period 2	Time period 3
**Age (years)**	26.8 (25.3 - 28.4)	24.2 (22.9 - 25.6)	25.9 (25.2 - 26.6)

**ISS**	11.0 (9.9 - 12.1)	6.1 (5.5 - 6.7)	7.1 (6.8 - 7.4)

**PSS-1**	8.8 (8.4 - 9.2)	10.2 (10.0 - 10.4)	9.8 (9.7 - 9.9)

**PSS-2**	11.6 (11.5 - 11.7)	11.5 (11.4 - 11.6)	11.3 (11.2 - 11.4)

**In-field response time (hours)**	1.6 (1.2 - 1.9)	0.9 (0.8 - 1.0)	0.65 (0.6 - 0.7)

**Total evacuation time (hours)**	4.4 (3.8 - 5.0)	3.6 (3.3 - 3.9)	2.3 (2.2 - 2.4)

The results are expressed as means with 95% confidence intervals

### Life-support interventions enhancing survival

**Figure 4:**
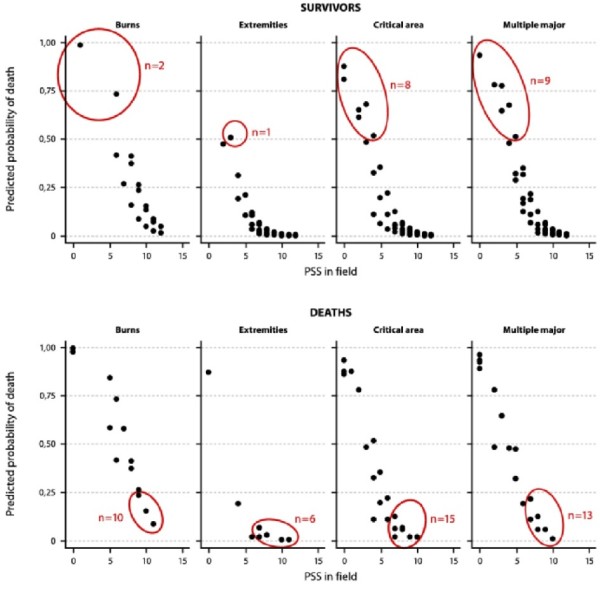
**Probabilistic model to identify unexpected survivors and unexpected fatalities**. In the scatter plot, survivors and fatalities are grouped by predicted probabilities of death, and physiological severity scores registered at the first in-field encounter (PSS 1). Red rings mark the unexpected survivors and unexpected deaths. Unexpected survivors were defined as survivors with higher than 50% risk of death according to the probabilistic model; unexpected deaths were defined as fatalities with less than 25% risk of death. "Critical area" implies injuries to the head, neck or the torso.

Diagnosis, category (blunt/penetrating), ISS, and PSS explained 77% of the variation in trauma mortality and gave a good fit with a ROC-AUC value of .99; ISS was the dominant predictor, alone yielding a ROC-AUC value of .98. Twenty seriously injured patients with ISS from 9 to 30 were identified as unexpected survivors, and there were 44 unexpected fatalities, all of them major trauma victims with ISS > 15 (Figure 4). In the group of unexpected survivors, all patients were in poor physiological condition at the first in-field encounter with a PSS ≤ 6 but had improving physiological indicators during the prehospital phase. Twelve patients with traumatic brain injury were among the unexpected fatalities with critical area injuries, all twelve dying within 48 hours after injury. These deaths occurred before neurosurgical service was established at the referral hospitals in 2006. Also in the group of unexpected fatalities were three cases with abdominal hemorrhage dying immediately on admission after two-hours' prehospital transit time. Six patients diagnosed as "extremity injury" suffered unexpected deaths due to associated head injuries. Among the 13 unexpected deaths with multiple major injuries, seven patients were admitted with close to normal physiological scores but died from internal hemorrhage in hospital hours after admission, one of them a patient with traumatic brain injury who did not undergo neurosurgery; four of the seven patients were injured by fragmentation mines. Ten burn fatalities with probability of death > 0.25 had PSS > 10 on admission but died within one week after the injury from infectious complications and/or organ failure.

There were 36 "prehospital treatment failures" defined as seriously injured on-site survivors with deteriorating out-of-hospital physiological severity scores despite care being provided. In eight cases diagnosed in the field as "extremity injury", limb bleeds were efficiently controlled but still the level of consciousness deteriorated during the prehospital phase due to undiagnosed brain injuries. In the other cases in the treatment-failure group, the main reason for deteriorating PSS values was worsening respiratory rate scores.

To identify specific life support measures with effect on survival, patients with respiratory problems and external bleeds were scrutinized. Most patients with airway problems were managed by basic measures only; endotracheal intubation was done only in 19 patients, crico-thyrotomy in one. Forty-seven patients had severe chest injuries with ISS ≥ 9. In this group, 39 patients had less than optimal respiratory scores in-field but 30 of the 39 had normal respiratory rate at end-point. Eighty-two patients with severe limb bleeds had BP < 70 mm Hg at first in-field; 69 of them were normotensive on hospital admission. The only fatal case in this group of patients was one man found three hours post-injury with traumatic double amputation from a fragmentation mine.

### Costs and effectiveness

Throughout the study period 180 paramedics were trained and joined the trauma system. By the end of 2006, 135 of them remained active. The treatment costs per patient (medical treatment, evacuation, data gathering and quality control) varied during the study period from US$ 130 to US$ 180.

## Discussion

The trauma system worked well, outcomes improving by time. Adjusted for severity alterations during the study period there was a significant reduction in mortality rates in critical area and multiple major injuries, except for burns. Rising incidence of self-inflicted burns in young women in certain feudal districts after the 2003 invasion account for increased mortality rate in burns observed in study period 3. The time from injury to first paramedic encounter in the field decreased during the study period. In-field response time is a risk factor for trauma death in major trauma victims; short paramedic response time is thus another indicator of better system quality. The actual study did not examine the first-responder impact, but a recent study of the same cohort demonstrated that early first aid by lay first responders contributes to improved survival [13].

There are several limitations to the study. Firstly, for ethical reasons the study was conducted without case-controls; selecting control cases from the districts with established EMS would not comply with established guidelines: "Members of any control group should be provided with an established effective treatment, whether or not such treatment is available in the host country" [14]. One random effect of the time-cohort design was severity variations throughout the study period. The ISS is a sensitive predictor of trauma death and lower fatality rates in period 2 and 3 may partly be explained by lower incidence rates of severe injuries. However, adjusting for anatomical and physiological severity by regression analysis there was still a significant reduction of total and prehospital mortality rates by time cohort. Yet there may have been unmeasured variables such as variations in war weaponry and variations in the quality-of-training or the quality-of-care provided by paramedics, but we hold that such variables would have minor impact on trauma outcome compared to the very heavy death risk predictor ISS. Secondly, the prehospital variables are registered by non-graduate paramedics at the site of injury and during rough evacuations, no concurrent independent validation being possible. On the other hand, the paramedics were well trained in physiological trauma scoring, and the documentation in each and every case was scrutinized in retrospect at monthly meetings with the main author. Thirdly, there may be unregistered prehospital fatalities. According to prevailing religious beliefs, however, people who die should be found and buried as soon as possible. As the trauma system consists of health workers and volunteers rooted in the local communities, very few local accidents will escape their attention. Finally, the ISS grading of on-scene fatalities are based on clinical examination only; for religious reasons, autopsy was not done. Hence, severity grading in these cases was systematically conservative. In summary we hold that the observed reduction in trauma mortality is reliable despite contextual changes during the study period.

As children react to trauma differently from adults, a special severity-scoring index, the Pediatric Trauma Score (PTS), is developed [15]. In the actual study the PTS was not applied in pediatric victims but standard severity scoring indices for adults, ISS and PSS. ROC analysis of the ISS and PSS-accuracy in death risk prediction showed that these two scoring systems had high accuracy both in the pediatric subsample and in the adult subsample, ROC-AUC 0.91 and 0.98 respectively. Also other studies of pediatric trauma victims confirm that the RTS is at least as sensitive as the PTS in identifying major pediatric trauma victims [16]. For this reason the pediatric trauma patients were not analyzed as a separate subsample in the actual study. The finding may have implications for Trauma Registry set-up in general; using the same severity scales across age groups makes things simpler with less risk of registration failures.

### Trauma audit

The high rate of unexpected deaths, 25% of all fatalities, should concern us; were these deaths avoidable? Some of the unexpected deaths from traumatic brain injuries could probably have been avoided if neurosurgical service had been in place throughout the study period. Most of the unexpected deaths in patients with abdominal bleeds might have been avoided if damage control surgery had been conducted at an early stage at a district hospital or immediately on admission at the referral hospital. The effect of the prehospital treatment was good also in burn cases; however, this did not have any significant impact on burn fatality rates, which remained high throughout the study period. Most burn fatalities, including the ten unexpected deaths observed in the study, are late deaths due to postinjury immune depression; in such cases survival depends on postinjury surgical care rather than prehospital life support. We should thus conclude that there is ample room for improvement of in-hospital trauma care in the study area.

Six patients diagnosed by the paramedic as "extremity injury" suffered unexpected deaths. In these patients the level of consciousness deteriorated during the prehospital phase despite efficient control of the external bleeding. The findings indicate that associated injuries (traumatic brain injury, internal hemorrhage) went undiagnosed by the paramedic. Especially in high-energy blast injuries (car bombs, fuel-air explosives) early clinical signs of brain injury and abdominal bleeds may be discrete and easy to miss [17]. We therefore recommend triage training especially for such mass casualties to help reduce miss-triage on-site and in the emergency room.

The prehospital treatment protocol is under debate and several studies question the usefulness of advanced measures [18]. Uncontrolled extremity bleeding is still a leading cause of avoidable battlefield deaths despite homeostatic agents are now being applied on wide scale in advanced trauma systems [19]. The actual simple treatment protocol - no tourniquet but sub-facial packing plus compression plus hypothermia prevention - proved effectual: 84% of extremity injured patients with severe in-field hemorrhage were normotensive on admission. We emphasize hypothermia prevention including warm IV fluids as part of the in-field treatment protocol for bleeds. In the actual study we did not gather data on core temperature, but previous studies conducted in the same study area document significant impact of simple preventive measures on body core temperature through protracted evacuations [20]. Airway block in unconscious patients is another common reason for avoidable trauma death. Very few study patients (< 1%) received advanced airway support in-field. Of four prehospital deaths from traumatic brain injury, one might have been prevented by in-field tracheal intubation; in the group of non-head injured unconscious patients we could not identify any preventable deaths caused by airway block. The findings indicate that basic airway measures are sufficient to control the airway in most risk cases. The treatment protocol did not included in-field chest tube drainage. There was one prehospital chest fatality, a patient with large chest wall wound. Among the other severe chest cases 75% had normal respiratory rate on hospital admission. Also for chest injured it seems that basic life support measures done early is the key to survival - IV ketamine pain relief, half-sitting position, and hypothermia prevention.

The intervention had a sustained impact on the quality of the EMS system in the study area. Despite adverse working conditions the overall retention rate of trained paramedics was high, 75%. The system performed on low costs; per-case costs of less than US$ 200 including systematic quality control should be a feasible price for most low-income communities. Also on national scale the model has had an impact; a two-tier dispatch system is now under implementation in the major cities in North Iraq, and there are requests from the Ministry of Health to implement the actual chain-of-survival model also in Central and South Iraq.

## Conclusion

Rural prehospital trauma systems reduce trauma mortality. Where out-of-hospital times are long, basic life support measures by trained lay first helpers and paramedics are life saving. Outcomes would probably improve further if damage control surgery had been carried out at local and referral hospitals. Miss-triage on-site and in the emergency room of patients with multiple major injuries is another cause of avoidable deaths; triage training should especially target bomb blast casualties.

## Competing interests

The intervention and the study were funded by humanitarian grants from The Norwegian Ministry of Foreign Affairs. The funder set no restraints for data access or publication of findings. The authors have no conflicts of interest related to the actual study, and there are no copyright constraints.

## Authors' contributions

The intervention was conceived by HH, designed and implemented by MKM and HH. MKM gathered, validated, and processed the data. MKM, SL, and HH analyzed the data, wrote the text, and approved the final manuscript.
